# Applications of CRISPR Technology to Breast Cancer and Triple Negative Breast Cancer Research

**DOI:** 10.3390/cancers15174364

**Published:** 2023-09-01

**Authors:** Mariona Pont, Marta Marqués, Maria Alba Sorolla, Eva Parisi, Izaskun Urdanibia, Serafín Morales, Antonieta Salud, Anabel Sorolla

**Affiliations:** 1 Research Group of Cancer Biomarkers, Lleida Institute for Biomedical Research Dr. Pifarré Foundation (IRBLleida), Av. Alcalde Rovira Roure, 80, 25198 Lleida, Spain; mpont@irblleida.cat (M.P.); mmarques@irblleida.cat (M.M.); msorolla@irblleida.cat (M.A.S.); eparisi@irblleida.cat (E.P.); iurdanibia@irblleida.cat (I.U.); serafinmorales01@gmail.com (S.M.); asaluds@hotmail.com (A.S.); 2 Department of Medical Oncology, Arnau de Vilanova University Hospital (HUAV), Av. Alcalde Rovira Roure, 80, 25198 Lleida, Spain; 3 Department of Medicine, University of Lleida, Av. Alcalde Rovira Roure, 80, 25198 Lleida, Spain

**Keywords:** breast cancer, triple negative breast cancer, CRISPR, applications, clinical trials

## Abstract

**Simple Summary:**

The technique of Clustered Regularly Interspaced Short Palindromic Repeats (CRISPR) has revolutionised cancer research, including breast cancer and triple negative breast cancer (TNBC). By employing this technique, scientists can now better model these diseases, discover unknown genes that play a role in cancer progression, facilitate a more sensitive and earlier diagnosis of breast cancer and triple negative breast cancer (TNBC), and even determine if there is the possibility of providing more selective and efficient treatments. To do so, scientists are trying to optimise the distribution of the CRISPR components in the tumour by using several methods that we have listed. In this work, we have also highlighted the weak points and the future perspectives that CRISPR possesses. Undoubtedly, CRISPR technology can improve many aspects of breast cancer/TNBC research.

**Abstract:**

Clustered Regularly Interspaced Short Palindromic Repeats (CRISPR) technology has transformed oncology research in many ways. Breast cancer is the most prevalent malignancy globally and triple negative breast cancer (TNBC) is one of the most aggressive subtypes with numerous challenges still to be faced. In this work, we have explained what CRISPR consists of and listed its applications in breast cancer while focusing on TNBC research. These are disease modelling, the search for novel genes involved in tumour progression, sensitivity to drugs and immunotherapy response, tumour fitness, diagnosis, and treatment. Additionally, we have listed the current delivery methods employed for the delivery of CRISPR systems in vivo. Lastly, we have highlighted the limitations that CRISPR technology is subject to and the future directions that we envisage. Overall, we have provided a round summary of the aspects concerning CRISPR in breast cancer/TNBC research.

## 1. Introduction

### 1.1. The CRISPR System: How It Works

Clustered Regularly Interspaced Short Palindromic Repeats (CRISPR) is a natural adaptative immune system present in prokaryote organisms such as bacteria and archaea. It protects bacteria against exogenous DNA such as viruses and plasmids that enter the cell. Such a defence mechanism relies on small, repeated nucleotide sequences (24–48 base pairs) flanked by DNA fragments called spacers. These PAM (protospacer adjacent motif) spacer regions have been incorporated into the bacteria genome in each of the previous infections. CRISPR genes are very well conserved and are associated with CRISPR-associated (Cas) genes. Cas protein processes the CRISPR sequences to produce the CRISPR RNA (crRNA) in order to eliminate the DNA molecule of the invader organism. These molecules are complementary, allowing the hybridisation of both RNA sequences and blocking the action of the invader. The Cas nuclease degrades this duplex so that the mechanism of virulence is halted, and the cell is protected. In addition, the Cas protein is responsible for adding the PAM sequence after the infection to generate the “memory” of the immunity. Therefore, when an external agent infects the bacteria, the system is expressed and the agent can be neutralised [1]. 

There are three types of CRISPR-Cas systems: Type I CRISPR-Cas, Type II CRISPR-Cas, and Type III CRISPR-Cas. Type I and III contain specialised Cas endonucleases that process the crRNA. Once mature, each crRNA assembles into a large multi-Cas protein complex capable of recognising and cleaving nucleic acids complementary to the crRNA. In contrast, Type II CRISPR-Cas requires a trans-activating CRISPR RNA (tracrRNA) complementary to the repeated sequences of crRNA. The ribonucleoprotease RNAse III, in the presence of Cas9, processes the crRNA. In this case, the Cas enzyme is only responsible for crRNA-guided silencing. 

**Figure 1:**
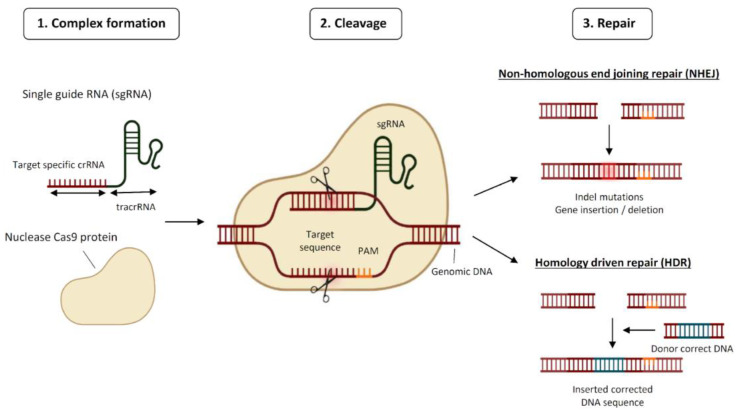
Schematic representation of the mode of action of CRISPR. Abbreviations: PAM, protospacer adjacent motif; tracrRNA, trans-activating CRISPR RNA.

CRISPR technology has revolutionised the field of genetic engineering by enabling localised gene disruption. Its use as a genome editing tool emerged a decade ago and today there is a long list of applications across cell types and organisms [2,3]. Currently, the Type II CRISPR-Cas system from *Streptococcus pyogenes* (spCas9) is the most used one. It is composed of a complex formed by the conjugation of the crRNA (the targeting molecule), the tracrRNA (the scaffold), and the Cas9 protein (the effector). Altogether, it recognises the target DNA near the PAM sequence, being the PAM sequence 5′-NGG-3′, where “N” can be any nucleotide base, and cuts 3–4 nucleotides upstream the PAM producing a double-strand break (DSB) [4]. The cell then tries to correct the DNA damage following one of the two main pathways, the non-homologous DNA end joining (NHEJ) pathway and the homologous direct recombination (HDR) pathway [5] (Figure 1). The NHEJ pathway introduces an indel that commonly interrupts the open reading frame of the gene, inducing a knockout. The HDR pathway has higher fidelity and is only functional during the S/G2 cell cycle phases. Through the HDR mechanism, it is also possible to provide foreign DNA to generate a knock-in. Besides Type II CRISPR-Cas with spCas9, there are other improved Cas variants including Type V CRISPR-Cas12a (formerly Cpf1) which is smaller than spCas9 [6], or Type VI CRISPR-Cas13 which is able to cleave single-stranded RNA [7]. Finally, an interesting modality of Cas9 is the deactivated Cas9 (dCas9), which harbours two mutations at its RuvC1 and HNH nuclease domains that leaves the enzyme catalytically inactive. This variant acts as a carrier of transcriptional regulators and performs epigenetic editing at the genomic region of interest [8].

### 1.2. Breast Cancer/Triple Negative Breast Cancer: Incidence, Genetic, and Epigenetic Alterations

Breast cancer is the most common cancer diagnosed worldwide and the most frequent cause of cancer death in women followed by lung cancer [9]. Furthermore, breast cancer is the fifth leading cause of cancer mortality worldwide considering both sexes [10].

Breast cancer can be classified in different types according to the presence or absence of oestrogen receptor (ER), progesterone receptor (PR), and the human epidermal growth factor receptor 2 (HER2) gene-overexpression/amplification. Considering this, breast cancer can be grouped as hormone receptor-positive (ER+ and/or PR+), HER-2 enriched (HER2+) when there is overexpression/amplification of this gene, or triple negative, which is deficient in the expression of all markers (ER−, PR−, HER2−) [9]. 

Approximately 15 to 20% of all breast cancers are triple negative breast cancers (TNBCs). TNBC is most common in premenopausal women younger than 40 years, and it has a poor prognosis, high invasiveness, high metastatic behaviour, high risk of recurrence, and lacks targeted therapies compared with hormone receptor-positive and HER2-enriched breast cancers. Generally, patients with TNBC have a shorter survival time and almost 46% of TNBC patients will develop distant metastasis, often in the lungs, liver, and brain [9]. Regarding the treatment, TNBC is not sensitive to hormonal therapy or current targeted therapies. Chemotherapy is therefore the mainstay treatment option but its efficacy is low once TNBC has progressed to metastasis [11]. 

Regarding genetic alterations in TNBC, the main and most common alterations of this type of breast cancer are in the genes *TP53*, *Breast Cancer 1/2* (*BRCA1/2*), *Phosphatase and tensin homolog* (*PTEN*), *Phosphoinositide-3-kinase catalytic subunit* (*PI3KCA*), *MYC*, *Epidermal growth factor receptor* (*EGFR*), *Engrailed 1* (*EN1*), and *Forkhead box C1* (*FOXC1*), among others [12,13].

The most frequently mutated gene in TNBC is *TP53*, which is altered in more than 80% of the TNBC tumours in the form of deletion or insertion [13]. Mutations in this gene result in higher genetic instability, higher metastatic risk, and worse overall survival. Another relevant gene is *BRCA1/2*, which plays an important role in DNA double-strand break repair and DNA stability. It has been observed that the *BRCA1* gene can be inactivated epigenetically in TNBC by promoter methylation, conferring poor prognosis [14,15]. Regarding the Phosphoinositide-3-kinase (PI3K)/AKT/Mammalian target of rapamycin (mTOR) pathway, TNBC commonly presents *PTEN* mutations, whereas mutations in *PI3KCA* are less frequent. *PTEN* mutations confer loss of expression, causing an increase in tumour cell proliferation and poor prognosis. Also, tyrosine kinase receptors such as EGFR receptors have been seen to be overexpressed in almost 50% of TNBC cases, while *EGFR* amplifications or high copy number variations are less common [15]. 

Overexpression of transcription factors has also been associated with the pathogenesis of TNBC. One case is EN1, where its overexpression is related to the activation of prosurvival pathways and the acquisition of resistance to chemotherapy [16]. Another transcription factor is MYC, which is overexpressed in almost 50% of TNBC. Moreover, it has been described that 45% of the *BRCA1*-mutated TNBCs also harbour *MYC* amplification. Finally, the transcription factor *FOXC1* is highly overexpressed in TNBC, where it has been reported to induce survival, proliferation, epithelial-to-mesenchymal transition (EMT), metastasis, invasiveness, and chemoresistance [14,17,18]. 

**Figure 2:**
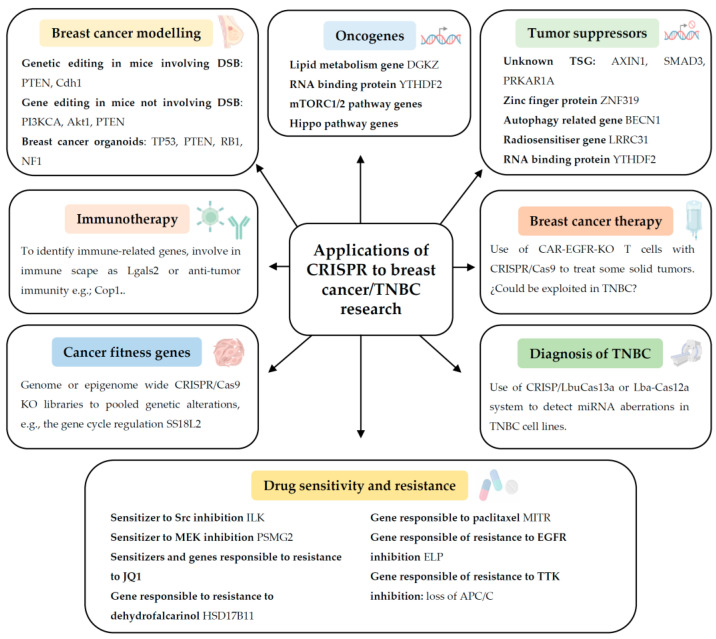
Summary of the applications of CRISPR technology to breast cancer and triple negative breast cancer (TNBC) research. Abbreviations: Akt1, RAC(Rho family)-alpha serine/threonine-protein kinase 1; APC/C, anaphase-promoting complex/cyclosome; BECN1, beclin1; CAR-T, chimeric antigen receptor T; Cas, CRISPR-associated; Cdh1, Cadherin 1; Cop1, COP1 E3 ubiquitin ligase; DGKZ, diacylglycerol kinase zeta; DSB, double-strand break; EGFR, epidermal growth factor receptor; ELP, Elongator complex; HSD17B11, 17β-hydroxysteroid dehydrogenase type 11 enzyme; ILK, integrin-linked kinase; KO, knockout; Lba, *Lachnospiraceae bacterium*; Lbu, *Leptotrichia buccalis*; Lgals2, galectin 2; LRRC31, leucine rich repeat containing 31; MEK, mitogen-activated protein kinase-kinase; mTORC1/2, mammalian target of rapamycin complex 1/2; MITR, MEF2-interacting transcriptional repressor; NF1, neurofibromatosis type 1; PI3KCA, phosphatidylinositol 3-kinase catalytic subunit; PRKAR1A, protein kinase cAMP-dependent type I regulatory subunit alpha; PSGM2, proteasome assembly chaperone 2 protein; PTEN, phosphatase and tensin homolog; RB1, retinoblastoma 1 gene; T cell, lymphocyte T cell; SMAD3, mothers against decapentaplegic homolog 3; Src, proto-oncogene tyrosine-protein kinase Src; TP53, tumour protein p53; TSG, tumour suppressor gene; TTK, threonine tyrosine kinase; YTHDF2, YTH N6-methyladenosine RNA binding protein F2; ZNF319, zinc finger 319.

Apart from these known genes, there are still relevant oncogenes pending to be discovered. In this review, we will focus on different recent genome-wide CRISPR/Cas9 knockout (KO) screenings that have been applied to identify critical oncogenes, tumour suppressor genes, genes responsible for drug resistance/sensitivity, immunotherapy response, and gene fitness in breast cancer or TNBC. We will also discuss the applications of CRISPR technology for early TNBC diagnosis, and breast cancer therapy (Figure 2). Lastly, we will highlight the current limitations and future perspectives of CRISPR regarding breast cancer/TNBC research.

## 2. Applications of CRISPR to Breast Cancer/TNBC Research

### 2.1. Modelling TNBC Genetically in Cells, Organoids, and Animals

The high heterogeneity present in breast cancer makes modelling of the disease quite difficult. New approaches to create a flexible platform that can be used to recapitulate genetic events, that are clinically relevant, are needed. CRISPR technology can constitute a valuable tool to model breast cancer/TNBC genetically in cells, organoids, and animals.

The CRISPR/Cas9 platform has been used to induce somatic mutations in vivo to model breast cancer and TNBC. To do so, a research group has followed two strategies. In breast cancer, they performed intraductal delivery of lentiviral vectors encoding for the Cre recombinase and the CRISPR/Cas9 system [19], or delivery of single guide RNAs (sgRNA)-encoding vectors [20]. In the first work, the authors were able to model invasive lobular breast carcinoma by sgRNA-assisted KO of *PTEN*, in female Cas9-knock-in and tissue-specific KO mice for the *Cdh1* gene, encoding for E-cadherin [19]. In the second work, with the intention of physiologically modelling TNBC better by installing precise point mutations while avoiding DSB, the authors made use of base editors. In particular, they utilised a knock-in mouse with Cre-conditional expression of the BE3 cytidine base editor able to produce C-to-T transitions within defined windows of the protospacer. The intraductal delivery of lentiviral vectors encoding sgRNAs, together with the base editor, was designed to generate missense mutations in *PI3KCA* and *Akt1*, and nonsense mutations in *PTEN* in mice lentivirally overexpressing *Myc*. Such a combination of genetic alterations increased the TNBC tumour burden [20].

However, in some cases, mice do not constitute a strong cancer model. For a deeper approach regarding gene screening and recapitulation of some clinically relevant mutations, the generation of human breast organoids could be a useful substitute. Dekkers et al. successfully modelled ER-positive breast cancer tumours responsive to chemotherapy and immunotherapy by inducing targeted KOs of *TP53*, *PTEN*, *Retinoblastoma gene* (*RB1*), and *Neurofibromatosis type 1* (*NF1*) with specific CRISPR/Cas9 sgRNAs in organoids. Organoids of different epithelial subtypes were established by the combination of basal progenitor cells CD49f^high/EpCAMlow^, luminal progenitor cells CD49f^low/EpCAMhigh^, and CD49f^low/EpCAMhigh^ mature luminal cells [21].

### 2.2. Identification of Novel Oncogenes in TNBC

CRISPR can serve for the identification of TNBC driver oncogenes. In this regard, Zhao et al. generated a CRISPR/Cas9 KO library containing lipid metabolic genes and identified *Diacylglycerol kinase Z* (*DGKZ*) as a potential metastatic candidate gene [22]. After performing a KO of *DGKZ* in TNBC cell lines, they found that it inhibits metastasis in vitro and in vivo, whereas its overexpression increased the metastatic capability of the cell lines. All their findings indicated that DGKZ could be used as a potential prognostic biomarker in patients with TNBC [22].

In the same line, in another study conducted by Einstein et al., the authors performed a pooled CRISPR/Cas9 screening by using a CRISPR/Cas9 lentiviral library targeting more than 1000 RNA-binding proteins in the human genome [23]. They identified different proteins required for the survival of *MYC*-driven breast cancer cells, where TNBC is an example. Specifically, the authors defined that the depletion of the RNA-binding protein YTHDF2 induced apoptosis in human TNBC cell lines and blocked the growth of TNBC MDA-MB-231 xenografts in vivo. Moreover, they described that this protein contributes to EMT and tumorigenesis. All this together defined *YTHDF2* as a tumoral promoter gene that could be considered as an effective therapeutic target [23].

In another work, conducted by Dai et al., the authors performed a genome-wide loss-of-function CRISPR/Cas9 screen in TNBC SUM159PT xenografts [24]. There, they identified positively and negatively selected genes whose inhibition enhanced or blocked tumour growth, respectively. Further experiments proved an existing cooperation between the mTORC1/2 pathway and the Hippo pathway for TNBC tumour progression. This was also reflected by the therapeutic synergistic interaction observed with two inhibitors of these pathways, verteporfin and Torin1, in reducing TNBC in vivo tumour growth [24].

### 2.3. Identification of New Tumour Suppressor Genes in Breast Cancer in General

Another application of genome-wide CRISPR/Cas9 screening consists of the identification of new tumour suppressor genes having a role in breast cancer progression and sensitivity to radiation therapy. 

In this line, Heitink et al. performed an in vivo CRISPR/Cas9 screening using primary mouse mammary epithelial cells and found different tumour suppressors involved in breast cancer [25]. They performed genetic engineering of both primary mammary organoids and in vivo models and identified some well-known tumour suppressor genes such as *PTEN*, *RB1,* and *NF1* and others such as *AXIN1*, *SMAD3*, or *PRKAR1A* that, when mutated, collaborate with *TP53* loss during mammary tumorigenesis. Another study conducted by Wang et al. identified a member of the zinc finger protein family, *ZNF319*, as a novel metastasis suppressor gene [26]. After performing a genome-wide CRISPR/Cas9 KO screen in an orthotopic breast cancer MCF7 xenograft, they found that *ZNF319* could be implicated in the regulation of breast cancer progression. Gene reconstitution experiments halted TNBC growth and invasion in vitro and in vivo. Moreover, high expression of ZNF319 in breast tumour tissue correlated with a better clinical outcome. Similarly, Wijshake et al. investigated the mechanism through which *BECN1* promotes tumour suppression by performing a loss-of-function, genome-wide CRISPR/Cas9 in the MCF7 cell line [27]. The screening results suggested that the loss of two members of the E-cadherin complex, *CDH1* and *CTNNA1*, could be mediators of the *BECN1*-mediated tumour suppressor mechanism. Regarding gene dependencies, Oser et al. performed a CRISPR/Cas9 screen in the small cell lung cancer cell lines NCI-H82 and NCI-H69 lacking *RB1*, to identify synthetic lethal mechanisms triggered by RB1 loss [28]. The authors discovered that *RB1*-deficient cells were highly dependent on Aurora B kinase for survival, suggesting that RB1 loss could be a good predictive biomarker for sensitivity to Aurora B kinase inhibitors. Indeed, the Aurora kinase inhibitor AZD2811 induced more mortality in the *RB1*-deficient breast cancer cell lines BT549 and DU4475 than in the proficient ones. Regarding gene cooperation networks, Zhao et al., performed a CRISPR/Cas9 screening coupled with single-cell RNA sequencing in genetically engineered human mammary epithelial MCF10A cells (MCF10-PTEN^-/-^, MCF-PI3KCA, and MCF10A-MYC) to identify how different driver genes could cooperate and how the combination of inactivated tumour suppressor genes could have an effect on the oncogenic properties and the transcriptome of breast epithelial cells [29]. The profiling of the genetic interactions indicated that different tumour-promoting genes, *PTEN*, *NF2*, *TP53*, *SMAD4,* and *CBFB,* shared related functions. They found that *PTEN* inactivation drives malignity in MCF10-PI3KCA cells, which is consistent with the co-occurrence of *PTEN* and *PI3KCA* alterations in breast cancer. The authors also discovered that transcriptional epistasis was the mechanism by which approximately 50% of the cancer driver genes cooperated to promote tumourigenicity in breast cancer.

Regarding ionising radiation, Chen et al. performed a loss-of-function, genome-wide CRISPR/Cas9 screen with an sgRNA library using MDA-MB-231-Br-HER2 cells, which fairly reproduced breast cancer brain metastasis, to find radiosensitiser genes [30]. After irradiating cells with a lethal dose (10 Gy), they identified *LRRC31.* Accordingly, *LRRC31* downregulation increased radioresistance and *LRRC31* overexpression sensitised cells to radiation. Mechanistically, they found that LRRC31 interacted with Ku70/Ku80 and ataxia telangiectasia mutated (ATM) and RAD3-related (ATR) to inhibit DSB. 

### 2.4. Identification of Genes Responsible for Immunotherapy Response in TNBC

CRISPR/Cas9 genetic screens have also been utilised for the identification of genes responsible for immunotherapy success and failure, and immunosuppression in TNBC. However, the number of articles is rather limited.

In an attempt to identify genes causative of immunosuppression in TNBC, Ji et al. generated a CRISPR/Cas9 sgRNA library in mice targeting all human disease-related immune genes [31]. *Lgals2* was pulled out from the screen as a promoter of the immune escape mechanism consisting of an increase in tumour-associated macrophages and their switch to the prooncogenic phenotype M2-like. Likewise, *Lgals2* overexpression and inhibition via CRISPR/Cas9 edition increased or reduced in vivo tumour growth, respectively, suggesting that *Lgals2* could be a promising target for immunotherapy. Another study that designed CRISPR/Cas9 sgRNAs targeting 4500 different genes implicated in tumour initiation, progression, and immune modulation in TNBC 4T1 cells implanted into syngeneic aimed to identify new immune targets regulating the tumour microenvironment. The authors discovered that the deletion of E3 ubiquitin ligase *Cop1* sensitised TNBC 4T1 tumours as well as the murine tumour models EMT6 (TNBC) and MC38 (colon) to immunotherapy. The mechanism proposed was through a decrease in macrophage infiltration by the regulation of macrophage-associated chemokines [32].

### 2.5. Identification of Genes Responsible for Drug Sensitivity and Resistance in Breast Cancer and in TNBC

Genome-wide CRIPSR/Cas9 KO screens have had a decisive role in identifying genes and pathways responsible for drug sensitivity or cytotoxicity, from known drugs to experimental therapies, in breast cancer in general and in TNBC. In TNBC, for instance, Beetham et al. performed a CRISPR/Cas9 screening in MDA-MB-231 cells to identify genes whose decrease altered the cellular sensitivity to the Src inhibitor bosutinib [33]. The study revealed that the loss of integrin-linked kinase (ILK) in combination with Src inhibition could be a new opportunity for increasing the clinical effectiveness of Src in TNBC [33]. In another study, Wang et al. pulled out the proteasome assembly chaperone 2 protein (PSMG2) from a genome-wide CRISPR/Cas9 screen as a sensitiser to the MEK inhibitor AZD6244. Similarly, the combination of a proteasome inhibitor with a MEK inhibitor synergistically inhibited cancer growth in TNBC cell lines and in the 4T1 xenograft, which could be used as an effective therapy [34]. Moreover, Shu et al. performed a genome-wide CRISPR/Cas9 KO screen combined with a small molecule inhibitor screen in the TNBC cell lines SUM149 and SUM159 treated with the BET bromodomain inhibitor JQ1 and its resistant derivatives for the identification of synthetic lethal or resistance interactions with JQ1. The resulting genes were classified into different functional categories. The deletion of genes involved in the kinase signalling pathway increased JQ1 responsiveness, whereas the ones involved in ubiquitination enhanced JQ1 resistance [35].

In a similar way but in normal breast cells, Barkovskaya et al. performed a CRISPR/Cas9 KO-mediated loss-of-function screen to find therapeutically exploitable vulnerabilities specific to both epithelial and mesenchymal phenotypes [36]. The authors discovered that epithelial-like cells were more sensitive to the loss of genes related to EGFR-RAS-MAPK signalling, while the mesenchymal ones were more sensitive to G2-M cell cycle regulator genes. Moreover, they suggested that EGFR inhibitors (gefitinib) and G2-M inhibitors could be a useful therapy to treat the epithelial and mesenchymal phenotypes, respectively. 

In TNBC, CRISPR/Cas9 screening has also been used to identify the mechanisms of cytotoxicity of natural compounds in TNBC. Grant et al. performed a CRISPR/Cas9-mediated gene KO screen to decipher the cytotoxic mechanism of dehydrofalcarinol, a compound obtained from the plant *Desmanthodium guatemalense* [37]. They identified *HSD17B11* (17β-hydroxysteroid dehydrogenase type 11 enzyme) to be responsible for the sensitivity to dehydrofalcarinol in MDA-MB-231 cells. 

Concerning drug resistance in TNBC, Lian et al. found, by employing a CRISPR/Cas9 screen in TNBC cell lines (MDA-MB-231 and BT-549) and in a MDA-MB-231 xenograft model, that the truncated isoform of histone deacetylase 9 (HDAC9), also known as MEF2-interacting transcriptional repressor (MITR), could be responsible for paclitaxel resistance [38]. In addition, by using an in vitro genome-wide single gene KO screen, Cruz-Gordillo et al. discovered that the Elongator (ELP) complex caused resistance to the EGFR inhibitor erlotinib in TNBC cells through the upregulation of the anti-apoptotic protein Mcl-1 [39]. Indeed, the pharmacological inhibition of Mcl-1 and EGFR was synergistic [39]. Furthermore, Thu et al. performed CRISPR/Cas9 screens in TNBC cell lines to identify mechanisms of resistance to the TTK protein kinase inhibitor CFI-402257 and others [40]. The anaphase-promoting complex/cyclosome (APC/C) came up from the screen as a predictor of such drug responses. The deficiency of APC/C allowed cells to tolerate genomic instability caused by the spindle assembly checkpoint (SAC) inactivation, and resistance to TTKi appeared as a consequence.

### 2.6. Determination of Cancer Fitness Genes in TNBC

It has been demonstrated that CRISPR screens can be applied to in vitro and in vivo models to characterise gene fitness in TNBC tumours. In this line, Eirew et al. developed a quantitative approach to pooled genetic alterations in patient-derived xenografts (PDXs), by encoding single cell output from transplanted CRISPR-transduced cells [41]. They showed that gene fitness depends on the number of transplanted cell clones and the variability in clone sizes.

Not only genome-wide CRISPR/Cas9 KO libraries have been generated to determine gene fitness but also epigenome-wide CRISPR/Cas9 KO libraries. This is the case with the library EPIKOL, designed by Yedier-Bayram et al. to target epigenetic modifiers and their cofactors [42] to identify genes relevant for cancer fitness in the TNBC cell lines MDA-MB-231, SUM149PT, and SUM159PT, and in MDA-MB-231 xenografts. Differently from other epigenome-focused libraries, EPIKOL, apart from targeting chromatin modifier proteins such as writers, readers, and erasers, also targets genes encoding chromatin complex cofactors and structural components. From this screen, a novel gene, the cell cycle regulator *SS18L2,* was identified.

### 2.7. Diagnosis of Breast Cancer and TNBC

CRISPR technology can also be deployed for the diagnosis of TNBC. Aberrant microRNA (miRNA) expression is a common cancer alteration exploitable for cancer diagnosis. Shan et al. demonstrated the utility of a programmed *Leptotrichia buccalis* CRISPR/LbuCas13a system for miRNAs detection with high specificity and sensitivity (as low as 4.5 attomoles (1 attomole = 10^−18^ mole)) in some TNBC cell lines [43]. The principle consists of activating LbuCas13 with the miRNA of interest. which then cuts a small RNA reporter with quenched FAM with (BHQ1)-labelled poly-U RNA probe (FQ5U). This disrupts the fluorescence resonance energy transfer and releases abundant fluorescent signals. The platform has proven to be useful for the detection of miR-17, miR-10b, miR-21, and miR-155. Moreover, the applicability of the Cas13a/CRISPR RNA (crRNA)-based miRNA detection system was verified in serum samples and extracts of small RNA from breast adenocarcinoma tissues. 

In another study, in breast cancer in general, performed by Wang et al., the authors proposed a new mechanism to detect miRNAs by using a novel *Lachnospiraceae bacterium* CRISPR/Lba-Cas12a-based strategy namely Cas12a-SRC. Different from the traditional mechanism, Cas12a recruits its crRNA by self-processing the pre-crRNA repeats generated by target-responsive rolling circle transcription and then the transcleavage activity of the real-time assembled Cas12a-crRNA can be activated. The main advantages of the strategy employed are that it provides low background detection, more sensitivity and specificity, and is more accurate for the detection of different miRNAs. Additionally, the authors evaluated the precision of Cas12a-SCR at determining the amount of miR-21 in 5 ng of total RNA from MCF7, HeLa, and HEK293T cells, which was aligned with their quantitative real-time polymerase chain reaction results [44].

### 2.8. Breast Cancer Therapy 

Currently, there are no ongoing clinical trials employing CRISPR for breast cancer treatment. However, the use of CRISPR for the genetic engineering of Chimeric Antigen Receptor (CAR)-T cells or editing T cells has become a reality for blood malignancies, with results published for B cell leukaemia [45]. Additionally, at the time that we wrote this manuscript, there were 12 ongoing clinical trials for leukaemia, lymphoma, and multiple myeloma (https://clinicaltrials.gov accessed on 26 April 2023). Despite such a success, it has not yet been possible to achieve positive effects in solid tumours or the clinical results are too preliminary [46]. This dose-escalation study assessed the dose and toxicity of the programmed death-1 (PD-1) and T cell receptor (TCR)-depleted CAR-T cell therapy in 15 patients with mesothelin-positive solid tumours, achieving stable disease in 2 patients. At the moment, there are four currently listed clinical trials in various solid tumours including renal cell carcinoma with results still to be reported. It is interesting to observe a clinical trial with solid tumours overexpressing EGFR (NCT04976218). This clinical trial is still in the recruiting phase; thus, there is a limited amount of information. The researchers involved exposed that one barrier to acquiring the efficacy of CAR-T cell therapy on solid tumours is the immunosuppressive role of the tumour microenvironment (TME). It has been known that transforming growth factor-β (TFG-β) is a significant regulatory factor in TME. This clinical trial aims to generate a CAR-EGFR-TGFβR-knockout T by deleting TGF-receptor II using CRISPR/Cas9, to study its anti-tumour activity and safety profiles in EGFR-positive advanced unresectable or metastatic biliary tract cancer, previously treated. It is conceivable that targeting EGFR could be exploited in the future for TNBC, given the prevalent overexpression of EGFR. 

## 3. Delivery Methods for CRISPR Technology for TNBC

A fundamental challenge that needs to be overcome to allow the clinical progress of CRISPR technology is the optimisation of its delivery, first in vitro and then in vivo, including humans. The delivery of CRISPR components has already been attempted since its inception and multiple methods have emerged. Depending on the nature of the delivery, CRISPR system delivery methods can be divided into three main categories: physical delivery, viral delivery, and non-viral delivery. In the group of physical methods, there are microinjection, electroporation, hydrodynamic tail vein injection (HTVI), ultrasonic microbubbles, and laser. The advantages of these methods are their high efficiency, simple operation, and low cost. Nonetheless, they present difficulties including being time-consuming. In the case of microinjection, it could be difficult to operate, and in regard to electroporation, the high voltage can cause cell damage and apoptosis. Finally, HTVI is nonspecific. Indeed, it has been observed that it can cause side effects in the liver and kidneys. Another CRISPR delivery method consists of the use of viral vectors which include lentiviruses, adenoviruses, and adeno-associated viruses. Their main advantage is the high efficiency of gene editing. However, viral vectors can elicit oncogenic effects, trigger immunogenic responses, and lead to insertional mutations. Lastly, the non-viral CRISPR delivery methods are characterised by having high delivery and editing efficiencies, low cost, and safety profile. However, some of them are quickly cleared when applied in vivo; they might be immunogenic and require a complex preparation process. Examples of non-viral CRISPR vehicles are polymers, liposome/lipid-based nanoparticles (NPs), gold NPs, other inorganic NPs, exosomes, cell-penetrating peptides (CPPs), and DNA nanoclews [47].

Regarding the in vivo CRISPR delivery in breast cancer or TNBC, the literature shows the usage of non-viral delivery methods in the format of nanoparticles, polymers or ribonucleoproteins, and viral vectors. The works are summarised in Table 1. 

Despite numerous studies on CRISPR delivery, it is noteworthy that there are some obstacles to achieving the implementation of CRISPR/Cas9 technology [58,59]. On the one hand, ensuring that the chosen system is safe and specific is important. More specificity would imply fewer off-target effects and higher safety involved. One way to overcome this obstacle is to assess the safety profile in the short but also long-term. Obtaining more information about where the components end up, how long they remain, and if any of them are toxic long-term, would be very meaningful. Overall, there has been a lot of promising research in the delivery of CRISPR, but further research is required to finally obtain an efficient, safe, and durable delivery of the CRISPR technology.

## 4. Limitations and Future Perspective of CRISPR Technology towards TNBC Research 

### 4.1. Extensive Use of In Vivo CRISPR Screens 

The use of genome-wide high-throughput CRISPR screens generated in vivo could broadly be implemented with the advancement of editing efficiency and cooperative research which will reduce cost and time. Either direct or indirect, they have multiple valuable applications for TNBC research as mentioned above. However, in vivo screens will have to cope with the increasingly imposed regulations against the use of animals for research by parliaments across the globe. This warrants extensive discussion between the implicated parties: scientists, policy-makers, and stakeholders, in order to find a balanced solution not detrimental to scientific advancement.

### 4.2. Direct Gene Editing in Breast Cancer Tissue 

The delivery of CRISPR components has been possible in breast cancer/TNBC animal models in mice [57], rats [60], and zebrafish [61], with the purpose of modelling and therapy. In terms of therapy, big progress has been achieved culminating with the first clinical trial in humans with refractory non-small cell lung cancer [62]. Since then, a list of human studies has followed, however, uniquely employing an indirect approach of in vivo gene editing which consisted of engineering allogeneic T cells. It is anticipated that direct gene editing of human breast cancer tissue with CRISPR will be a future possibility thanks to improvements in the efficiency of its in-target gene editing, reduction of off-target effects, and proper delivery. 

### 4.3. Novel Delivery Methods for CRISPR Technology

A myriad of delivery methods for CRISPR systems has emerged [47]. All of them possess advantages and disadvantages. The ideal vehicle for CRISPR delivery in vivo, especially for humans, will have to attain certain characteristics including ease of synthesis, biocompatibility, biodegradability, high packaging and encapsulation capacity, low immunogenicity, cellular type selectivity, high delivery efficiency, safety, and low cost. Delivery vehicles will evolve hand-by-hand with CRISPR technology methods to offer the best gene editing solution.

### 4.4. Modern Methods Coupled with CRISPR Technology

State-of-the-art methods such as artificial intelligence can be applied to sgRNA design to ensure the highest selectivity and specificity and to minimise off-target effects. Several works have outlined algorithms that have the potential to predict the effects of gRNAs on- and off-target, enhancing the specificity of the CRISPR/Cas9 system. Some algorithms are based on machine learning, a subset of artificial intelligence that uses existing datasets to anticipate the off-target effect in order to predict and optimise CRISPR gRNA in terms of activity and efficiency. These methods include CRISPRater (Heidelberg, Germany), CRISPRscan (New Haven, CT, USA), DeepCRISPR (Shanghai, China), and Azimuth 2.0 (Cambridge, MA, USA), among others [63]. The modern technique of single-cell sequencing can be coupled to CRISPR technology to track gene editing or gene expression of downstream effectors at the single cell resolution at a given period of time during breast cancer/TNBC progression. This can provide enormous information about the effects of a punctual genetic edition event. It can be utilised for generating single-cell CRISPR screens in breast cancer/TNBC cells, although they have a limited targeting power, only 100 genes.

### 4.5. Unequal Access to CRISPR Medical Advances and Other Ethical Aspects

Due to the fact that the application of CRISPR implies the modification of an organism’s genetic material, ethics concerns regarding the technique, application, use, purpose, and justice have been awakened [64]. Probably the least discussed issue is justice. The cost of a therapeutic intervention using CRISPR technology could range between USD 400,000 and USD 2 million, being out of reach for most patients and medical services worldwide. This can create a division in communities and impulse an elite class able to afford such treatments, lessening equality. This is known as aristocracy genetics. 

Apart from the economic issue, the possibility that CRISPR could be used for immoral practices such as enhancing a non-pathological trait, even passing over future legislation, is not inconceivable. This is the case of the scientist He Jiankui, who undertook genetic modification of embryos via CRISPR/Cas9 to deplete *CCR5* in order to lower their HIV infection risk. However, such a genetic alteration of the germinal line and embryos for intended reproduction was not permitted in China and the CRISPR-related off-target effects are still to be seen.

These ethical aspects of CRISPR technology will surely be a matter of intense debate in the future. They have already been questioned and exposed by various scholars in both the oral and written media.

## Figures and Tables

**Table 1:** Studies involving the in vivo delivery of CRISPR systems in breast cancer or triple negative breast cancer (TNBC) models.

Delivery Method	Subtype	Material	Applicability/ Delivery of	Target Gene/s	In Vivo Model of Breast Cancer/TNBC	Route and Frequency of Administration	Ref.
Non-viral delivery	Lipid-polymer hybrid nanoparticles	Phenylboronic acid—functionalised low molecular weight polyethyleneimine PEI 1.8k (PEI-PBA)	dCas9-based CRISPR interference system (CRISPRi)	*miR-10b*	4T1 TNBC allograft	Intravenously Every 5 days for 28 days	[48]
Non-viral delivery	Nanovesicles	Tumour-derived extracellular vesicles—fusogenic anthracycline doxorubicin liposomes (T-DOX)	CRISPR/Cas9	*PD-L1*	Orthotopic 4T1 TNBC allograft	Subcutaneously 1-day interval	[49]
Non-viral delivery	Nanobubbles	Polyethyleneimine (PEI)	CRISPR/Cas9	*Cdh2*	Orthotopic 4T1 TNBC allograft	N/A	[50]
Non-viral delivery	Polyethyleneimine–Bovine serum albumin-based nanoparticles	Polyethyleneimine– Bovine serum albumin (PEI-BSA)	CRISPR/Cas9 system in plasmid and ribonucleoprotein format	*CD81*	BALB/c mice	Intravenously One injection	[51]
Non-viral delivery	DNA-based nanoparticles	Polyglycerol Dimethacrylate	Co-delivery of Cas9/sgRNA ribonucleoprotein and DNAzyme	*PLK1* *EGR-1*	Breast cancer MCF7 xenografts	Intravenously Days 0 and 6	[52]
Non-viral delivery	Targeted core-shell nanoparticles	Polyacrylaminoester (PAA)	Dual plasmids pHR-pCas9	*CTCF*	Female BALB/c nude mice	Intravenously	[53]
Non-viral delivery	Nanoparticles	Polylysine functionalised black phosphorus (PLL-PBP)	PBP/Cas13a/ crMcl-1 complex	*Mcl-1*	TNBC MDA-MB-231 xenograft	Intratumourally Every two days for a total of 10 injections	[54]
Non-viral delivery	Autocatalytic brain tumour-targeted nanoparticles	HDL-DES-MDEA polymer	LRRC31 cDNA loaded NPs	*LRRC31*	Female BALB/c nude mice	Intravenously Days 6 and 11	[30]
Non-viral delivery	Polymeric nanoparticles	Poly-β-amino ester (PBAE)	aPBAE/cas9-Cdk5 complex	*Cdk5*	TNBC orthotopic 4T1 allograft	Intratumourally Days 7, 10, 13, and 16 post-inoculation of 4T1 cells	[55]
Non-viral delivery	Organic polymer	Poly-glycidyl methacrylate (PGMA)	CRISPR/dCas9 conjugated to the effector domains VPR or SAM	*MASPIN* *CCN6*	Breast cancer MCF7 xenograft	Intravenously Every 5 days	[56]
Non-viral delivery	Targeted nanolipogel (tNLGs)	Noncationic lipid bilayer and a biodegradable hydrogel core	Three CRISPR plasmids targeted to different DNA sequences of *Lnc2*	*Lnc2*	Orthotopic TNBC MDA-MB-231 xenograft	Intravenously Weekly, administered for 4 weeks	[57]
Viral delivery	Lentiviruses	Lentiviral pSECC vector encoding Cre and CRISPR components	sgRNA encoding vector	*PTEN*	Cas9-knock-in and mammary tissue-specific *Cdh1^F/F^* female mice	Intraductal injection	[19]
Viral delivery	Lentiviruses	Lentiviral	sgRNA encoding vector	*PI3KCA* *Akt1*	Mammary tissue specific of the base editor BE3 and Cre, Cas9 knock-in *Brca1^F/F^;Trp53^F/F^* female mice	Intraductal injection	[20]

Abbreviations: CRISPRi, clustered regularly interspaced short palindromic repeats interference; miRNA, microRNA; PD-L1, programmed death-ligand 1; SAM, synergistic activator mediator; sgRNA, single guide RNA; TNBC, triple negative breast cancer; Ref, reference; VPR, VP64-p65-Rta.
